# Comprehensive phenotypic assessment of nonsense mutations in mitochondrial ND5 in mice

**DOI:** 10.1038/s12276-024-01333-9

**Published:** 2024-11-01

**Authors:** Sanghun Kim, Seul Gi Park, Jieun Kim, Seongho Hong, Sang-Mi Cho, Soo-Yeon Lim, Eun-Kyoung Kim, Sungjin Ju, Su Bin Lee, Sol Pin Kim, Tae Young Jeong, Yeji Oh, Seunghun Han, Hae-Rim Kim, Taek Chang Lee, Hyoung-Chin Kim, Won Kee Yoon, Tae Hyeon An, Kyoung-jin Oh, Ki-Hoan Nam, Seonghyun Lee, Kyoungmi Kim, Je Kyung Seong, Hyunji Lee

**Affiliations:** 1 Department of Biomedical Sciences, Korea University College of Medicine, Seoul, 02841 Republic of Korea; 2 Laboratory Animal Resource and Research Center, Korea Research Institute of Bioscience and Biotechnology, Cheongju, 28116 Republic of Korea; 3 Laboratory of Developmental Biology and Genomics, Research Institute for Veterinary Science, and BK21 PLUS Program for Creative Veterinary Science Research, College of Veterinary Medicine, Seoul National University, Seoul, 08826 Republic of Korea; 4 Korea Model animal Priority Center, Seoul National University, Seoul, 08826 Republic of Korea; 5 Department of Physiology, Korea University College of Medicine, Seoul, 02841 Republic of Korea; 6 Metabolic Regulation Research Center, Korea Research Institute of Bioscience and Biotechnology (KRIBB), Daejeon, 34141 Republic of Korea; 7 Department of Functional Genomics, KRIBB School of Bioscience, Korea University of Science and Technology (UST), Daejeon, 34141 Republic of Korea; 8 Department of MetaBioHealth, Sungkyunkwan University (SKKU), Suwon, 16419 Republic of Korea; 9 Department of Precision Medicine, School of Medicine, Sungkyunkwan University (SKKU), Suwon, 16419 Republic of Korea; 10 Department of Convergence Medicine, Korea University College of Medicine, Seoul, 02708 Republic of Korea

**Keywords:** Genetic engineering, Energy metabolism

## Abstract

Mitochondrial dysfunction induced by mitochondrial DNA (mtDNA) mutations has been implicated in various human diseases. A comprehensive analysis of mitochondrial genetic disorders requires suitable animal models for human disease studies. While gene knockout via premature stop codons is a powerful method for investigating the unique functions of target genes, achieving knockout of mtDNA has been rare. Here, we report the genotypes and phenotypes of heteroplasmic MT-ND5 gene-knockout mice. These mutant mice presented damaged mitochondrial cristae in the cerebral cortex, hippocampal atrophy, and asymmetry, leading to learning and memory abnormalities. Moreover, mutant mice are susceptible to obesity and thermogenetic disorders. We propose that these mtDNA gene-knockdown mice could serve as valuable animal models for studying the MT-ND5 gene and developing therapies for human mitochondrial disorders in the future.

## Mitochondrial gene knockout in mice reveals brain abnormalities

Mitochondria, often referred to as cell's powerhouses, are essential for energy production and health. In this study, researchers used recently developed ways to edit mtDNA, allowing precise changes to study disease results in animal models. They performed an experiment involving the injection of specially designed mRNAs, into mouse embryos to cause specific changes in mtDNA. This method allowed the creation of mice with targeted mitochondrial gene removals, providing a useful model for studying mitochondrial diseases. Researchers found that mice with certain mtDNA changes showed symptoms like human mitochondrial diseases, such as brain abnormalities and metabolic issues. These findings emphasize the importance of mtDNA in health and disease, providing insights into how specific changes can affect bodily functions. Future research may use these findings to develop therapies targeting mtDNA changes. This summary was initially drafted using artificial intelligence, then revised and fact-checked by the author.

## Introduction

Mitochondria are essential organelles capable of producing chemical energy, and they play an important role in sustaining our survival. As energy powerhouses, mitochondria supply >90% of the adenosine triphosphate (ATP) necessary for cellular metabolism^1^. Additionally, mitochondria play crucial roles in various cellular processes, including the regulation of ion homeostasis, redox status, cell survival, and cell death^2^. Therefore, mitochondrial dysfunction caused by environmental damage, aging, and especially DNA mutations may contribute to various human diseases^3,4^.

While a cell’s DNA is located mainly in the nucleus, mitochondria contain their own DNA, known as mitochondrial DNA (mtDNA)^5^. mtDNA plays an essential role in cellular respiration and energy consumption in humans and mice^6^. It is a 16.5-kb circular double-stranded DNA that encodes proteins for the oxidative phosphorylation system, tRNAs, and ribosomal RNAs to aid in mitochondrial maintenance^7,8^. Unlike other organelles, mitochondria are enveloped by two distinct membranes, an outer membrane, and an inner membrane, with an intermembrane area between them^5^. Additionally, there are cristae (folds in the inner membrane), which are crucial for ATP production^9^, and a matrix, which is the space within the inner membrane. ATP produced in the mitochondria is synthesized by electron transferases in the inner membrane. These electron transferases include complex I (NADH/ubiquinone oxidoreductase), complex II (succinate dehydrogenase), complex III (cytochrome c reductase), complex IV (cytochrome c oxidase), and complex V (ATP synthase) in the mitochondrial cristae. Among them, mitochondrial complex I, which is composed of seven polypeptides (*MT-ND1, MT-ND2, MT-ND3, MT-ND4, MT-ND4L, MT-ND5*, and *MT-ND6*), is pivotal in the electron transport chain, a process essential for maintaining the biological system^10^. Therefore, mutations in complex I genes may lead to various human diseases^11^.

Mok et al. and Cho et al. successfully developed C-to-T and A-to-G base editing in the mitochondrial genome^12,13^, making it possible to induce programmable base editing in double-stranded mtDNA to demonstrate phenotypic changes in animals. Previous studies have recently established mice and rat models for mtDNA point mutations with distinct phenotypes, including hunched posture, brain anatomy abnormalities, mitochondrial morphology changes, and motor ability impairments^14,15^. Furthermore, heteroplasmic knockout of the mitochondrial gene, with a mutation load for *MT-ND5* gene knockdown of up to 46%, has been achieved^16^. However, comprehensive phenotypic assessments of mitochondrial gene knockouts have rarely been reported. Thus, this study investigated apparent phenotypes and genotypes as well as brain- and fat-related phenotypes in heteroplasmic mitochondrial gene-knockout mice throughout their generation.

## Materials and methods

### mRNA preparation

We used DdCBE-encoding plasmids from a previous study^17^. These mRNA templates were prepared via polymerase chain reaction (PCR) via Q5 High-Fidelity DNA Polymerase (M0492S, NEB, Ipswich, MA, USA) with the following primers: F: 5′-CATCAATGGGCGTGGATAG-3′ and R: 5′-GACACCTACTCAGACAATGC-3′. mRNAs were synthesized via an in vitro RNA transcription kit (mMESSAGE mMACHINE T7 Ultra kit, Invitrogen, Carlsbad, CA, USA) and purified via a MEGAclear kit (Invitrogen, Carlsbad, CA, USA). All procedures were conducted according to the manufacturer’s instructions.

### Animals

Hormone-injected wild-type (WT) C57BL/6 N female C57BL/6 N males were mated, and embryos were collected. Female mice from the ICR strain were used as surrogate mothers. Because mitochondria follow maternal inheritance, female mutant mice were used for breeding, and phenotype analysis was executed with male mutant mice. All the mice were maintained in a specific pathogen-free facility under a 12-h dark‒light cycle at a temperature of 20–26 °C and a humidity of 40–60%.

### Microinjection of mouse zygotes

In preparation for zygote microinjection, 4–6-week-old C57BL/6 N female mice were superovulated by injecting pregnant mare serum gonadotropin ([5 IU, Prospec, Ness-Ziona, Israel]) and human chorionic gonadotropin hormone (5 IU, Sigma‒Aldrich, Burlington, MA, USA) intraperitoneally at 48-h intervals. For microinjection, a mixture containing left and right DdCBE-encoding mRNAs (each at 300 ng/μL) was diluted in diethylpyrocarbonate-treated injection buffer (0.25 mM ethylenediaminetetraacetic acid [EDTA], 10 mM Tris; pH 7.4) and injected into the cytoplasm of zygotes via a Nikon ECLIPSE Ti micromanipulator and a FemtoJet 4i microinjector (Eppendorf, Hamburg, Germany). The embryos were then cultured in microdrops of KSOM + AA (Millipore, Burlington, MA, USA) at 37 °C for 2 days in a humidified atmosphere containing 5% carbon dioxide (CO_2_). Two-cell-stage embryos were then implanted into the oviducts of 0.5-dpc pseudopregnant surrogate mothers.

### Genotyping and targeted deep sequencing

All the assay methods used for genotyping were identical. Only the samples (blastocysts, toes, and feces) used as DNA templates for PCR differed. Blastocyst-stage embryos and tissues from pups were incubated in lysis buffer (25 mM sodium hydroxide [NaOH], 0.2 mM EDTA; pH 10) at 95 °C for 20 min, after which the pH was adjusted to 7.4 via the addition of 4-(2-hydroxyethyl)-1-piperazineethanesulfonic acid (HEPES, free acids without pH adjustment) at a final concentration of 50 mM. For targeted deep sequencing, nested first PCR and second PCR were performed to create a high-throughput sequencing library, after which the final index sequences were merged via Q5 DNA polymerase. The primers used are listed in Supplementary Table 1. The library was subjected to paired-end read sequencing via a MiniSeq platform (Illumina, San Diego, CA, USA). In all the cases, the sequencing results were joined into a single fastqjoin file and analyzed via the CRISPR RGEN tool (http://www.rgenome.net/).

### Oxygen consumption rates

The oxygen consumption rates were measured via a Seahorse XFe24 Analyzer (Agilent, Santa Clara, CA, USA) following the manufacturer’s protocol. One hundred microliters of suspended cells (10^6^ cells/ml) were seeded into Seahorse XF24 V28 PS cell culture microplates (Agilent, Santa Clara, CA, USA) 16 h before measurements were taken. An analysis was performed using Seahorse XF DMEM pH 7.4 supplemented with 25 mM glucose and 1 mM sodium pyruvate (Agilent, Santa Clara, CA, USA). XF cell mito stress tests were then performed using 1.5 mM oligomycin, 0.5 mM FCCP, and 0.5 mM rotenone + antimycin A (Seahorse XF Cell Mito Stress Test Kit 6 XF Assays, Agilent, Santa Clara, CA, USA).

### Western blotting & immunoblotting

Proteins were isolated from the brains of WT and mutant mice via a DNeasy Blood & Tissue Kit (QIAGEN, Venlo, Netherlands). The concentration of the cleared lysate was measured via a Bradford protein assay (Bio-Rad, Hercules, CA, USA). Prepared protein samples (total protein = 20 µg) were loaded onto SDS‒polyacrylamide gel electrophoresis (PAGE) gels with 6% acrylamide depending on the target protein and transferred onto polyvinylidene difluoride (PVDF) membranes (Bio-Rad, Hercules, CA, USA). These membranes were immunoblotted with the following primary antibodies: anti-mt-ND5 (PA5-36600, Thermo Fisher Scientific, Carlsbad, MA, USA), anti-NDUFB7 (NADH dehydrogenase [ubiquinone] 1 beta subcomplex subunit 7, sc-365552, Santa Cruz Biotechnology, Dallas, TX, USA), and anti-β-actin (SC-47778, Santa Cruz Biotechnology, Dallas, TX, USA). The membranes were subsequently incubated with the following secondary antibodies: anti-rabbit (7074, Cell Signal, Danvers, MA, USA) and anti-mouse (7076, Cell Signal, Danvers, MA, USA). Specific protein complexes were visualized via the Clarity Western ECL Substrate (Bio-Rad, CA, USA). The signals were detected with a ChemiDoc XRS+ system (Bio-Rad, Hercules, CA, USA). Band intensities were quantified by densitometry via ImageJ (NIH) and normalized to that of β-actin.

### Measurement of ATP concentration

ATP concentrations in the brain and liver were determined with an ATP Assay Kit (ab83355, Abcam, Cambridge, UK) according to the manufacturer’s protocol. Brain and liver tissues (10 mg) were used in the assay. Tissues were homogenized and lysed in ATP buffer. After lysis, the samples were centrifuged at 13,000 × *g* for 5 min at 4 °C to remove insoluble material. They were then loaded into a 96-well plate (SPL, Seoul, Korea) and diluted in ATP assay buffer at the recommended proportions following the manufacturer’s protocol. The plate was incubated at room temperature for 30 min in the dark. The optical density (OD) was then measured at 570 nm with a microplate reader.

### Transmission electron microscopy (TEM)

The tissues were dissected and fixed with 2.5% glutaraldehyde in 0.1 M phosphate buffer (pH 7.4) for 2 h at 4 °C, followed by postfixation with 1% osmium tetroxide for 2 h on ice. These tissues were then embedded in Epon 812 after dehydration in ethanol and propylene oxide. Polymerizations were performed using pure resin at 70 °C for 2 days. An ultramicrotome (UltraCut-UCT, Leica, Wetzlar, Germany) was used to obtain 70-nm ultrathin sections, which were collected on 100-mesh copper grids and stained with 2% uranyl acetate and lead citrate. The accelerating voltage for TEM (Technai G2 Spirit TWIN, FEI, Hillsboro, OR, USA) was 120 kV. Electron micrographs were obtained at a magnification of ×8000.

### Histological analysis

Mouse fat tissues were fixed in 10% formalin after perfusion with saline. Whole brains were soaked in 4% paraformaldehyde (PFA) solution for 5 days at 4 °C with shaking. The samples were embedded in paraffin and sectioned at a thickness of 5 μm. The sections were then stained with H&E. To visualize mid-brain structures and detect apoptotic markers, the sections were stained with antibodies against the neuronal marker NeuN (ab177487, Abcam, Cambridge, UK) and the apoptotic marker cleaved caspase-3 (9664S, Cell signal, Danvers, MA, USA). To investigate apoptotic regions in the hippocampus of a mutant brain, a terminal deoxynucleotidyl transferase dUTP nick end labeling (TUNEL) assay was conducted via a TUNEL assay kit (DeadEndTM Colorimetric TUNEL System, Promega, Madison, WI, USA) according to the manufacturer’s instructions. Briefly, the sections were permeabilized with 20 µg/ml proteinase K for 15 min and blocked with 30% hydrogen peroxide for 5 min at room temperature. Apoptotic signals were visualized via DAB, and hematoxylin was used as a counterstaining agent.

### Fear conditioning

Fear conditioning was used to evaluate aversive learning and memory. On Day 1, the mice were transported to a testing room and left undisturbed for 30 min before measurement. A tone cue at 80 dB was presented for 30 s, and a mild 0.8-mA foot shock was administered during the last 2 s of tone presentation. After an intertrial interval of 210 s, the procedure was repeated. On Day 2, the same light as that used on Day 1 was provided without tone cues. The mice were observed for the presence or absence of a freezing response. The acquired data were analyzed on the basis of International Mouse Phenotyping Consortium (IMPC) standards (www.mousephenotype.org) by tracking software (FZ; O’Hara & Co, Ltd., Tokyo, Japan).

### Open field test

The mice were placed in a 60 × 60-cm automated open field box that was homogeneously illuminated at 80 lx and allowed to explore freely for 20 min. Movement distance and speed in the arena were detected by a machine at 5-min intervals for 20 min and recorded via computer software. The acquired data were analyzed according to IMPC standards (www.mousephenotype.org) by tracking software (TimeOFCR4; O’Hara & Co, Ltd., Tokyo, Japan).

### Measurements of body weight, body composition, and body temperature

Body weight was measured on electronic scales (Sartorius, Gottingen, Germany) after zeroing at the same time every week. Body composition was determined via a dual-energy X-ray absorptiometer (DEXA, Lunar Piximus II, GE Medical Systems, Chicago, IL, USA). The mice were anesthetized with 1.2% avertin before measurement, and body temperature was measured via a rectal thermometer.

### Blood serum analysis

Glucose, total cholesterol, high-density lipoprotein (HDL), low-density lipoprotein (LDL), triglyceride, phospholipid, and phosphorous levels were measured via a HITACHI Automatic Analyzer 7020 (HITACHI, Ibaraki, Japan) following the manufacturer’s instructions. Samples for testing fasting blood sugar were obtained at 15–18 h after food restriction via retro-orbital blood collection. All blood samples were incubated at room temperature for 30 min and centrifuged at 12,000 rpm for 15 min to obtain serum samples.

### Indirect calorimetry

For indirect calorimetry assessment, the mice were housed separately in metabolic chambers (CLAMS double feeder cages, Columbus instruments, Columbus, OH, USA) and had free access to food and water. The oxygen consumption rates and CO_2_ production rates were measured for 24 hours. The metabolic data were analyzed simultaneously.

### Cold challenge

To assess the body temperature of the cold-exposed mice, the mice were exposed to 6 °C in a slow-temperature chamber (DHIN02-0034, DBL, Seoul, Korea). Body temperature was measured with a rectal thermometer (Testo 925, Testo, Kirchzarten, Germany).

### Whole mitochondrial genome sequencing

For whole mitochondrial genome sequencing, genomic DNA was extracted via a DNeasy Blood & Tissue Kit (Qiagen, Venlo, Netherlands). mtDNA was amplified via PCR via PrimeSTAR GXL polymerase (Takara, Shiga, Japan). Amplified mtDNA was subjected to tagmentation via a DNA prep kit (Illumina, San Diego, CA, USA) following the manufacturer’s protocol. The library was subjected to paired-end sequencing via the MiniSeq platform (Illumina, San Diego, CA, USA).

### Single-cell genotyping in organs

For single-cell genotyping, the organs were ground with a 10-ml syringe plunger, and the cells were divided using 40-μm cell strainers (93040, SPL, Seoul, Korea). Divided cells were added to DMEM (11965084, Gibco, Grand Island, NY, USA) and counted with a cell counter (AMQAX2000, Invitrogen, Carlsbad, CA, USA). After calculation, the cells were diluted and transferred to 96-well plates at 0.3 cells/well. Finally, individual cells were harvested, and genotyping was executed.

### Statistical analysis

The data are presented as the means $\pm \,$SEM for biologically independent samples, and *P* values were calculated via Student’s two-tailed *t* test. The following *P* values were used: *p < 0.05, **p < 0.01, and ***p < 0.0001. The statistical analysis was performed in Prism (GraphPad).

## Results

### Construction of *MT-ND5* heteroplasmic knockout mutant mice

**Fig. 1:**
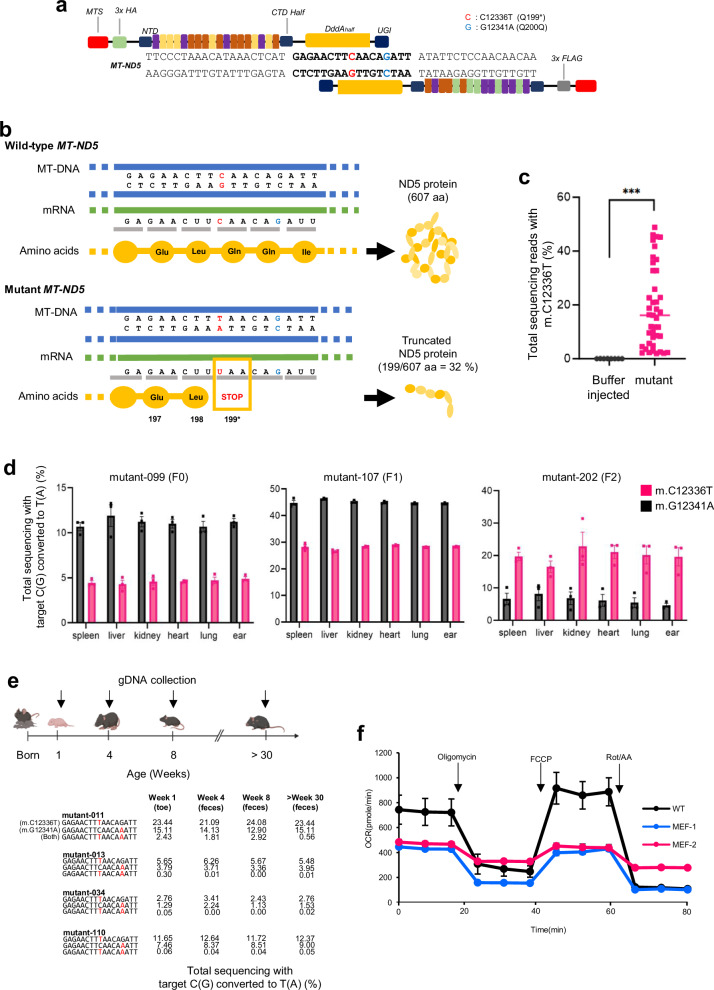
Generation of a heteroplasmic *MT-ND5* gene knockout mutant mouse model and genotyping. **a** Schematic illustration of DdCBE in the mouse *MT-ND5* gene. The base editing window is annotated in bold. Possible editing loci are shown in red (m.C12336T) and light blue (m.G12341A). Transcription activator-like effector (TALE)-binding arrays are depicted in different colors for each of the four nucleotides. **b** Diagram of designed base editing in *MT-ND5*. Base editing targets are indicated in red (m.C12336T) and light blue (m.G12341A). The nonsense mutation is introduced at the 199th amino acid. **c** Editing efficiency of m.C12336T in DdCBE-treated mice. Targeted deep sequencing data were obtained from the genomic DNA of the pups’ toes. The exact *p* value was 2.4E-11 (*p < 0.05, **p < 0.01, and ***p < 0.001 via two-tailed Student’s *t* test). **d** Efficiency of editing various tissues from F0, F1, and F2 mice (from the left). Genomic DNA was obtained from tissues at least 6 weeks after birth. The dark and light bars indicate the frequencies of m.C12336T and m.G12341A, respectively. The error bars indicate the standard error of the mean (s.e.m.) for replicates (*n* = 3). **e** Timeline of genomic DNA collection for targeted deep sequencing data and enumerations of mutant sequences from F0 and F1 newborn pups. Genomic DNA was isolated one week after birth from feces at four-week intervals and after 30 weeks. Edited bases are highlighted in red. **f** Oxygen consumption rates of mouse embryonic fibroblasts. Each respiratory parameter was normalized to the value of the WT. For MEF-1 and MEF-2, the m.C12336T-editing efficiencies were 19% and 21%, respectively. The error bars indicate the SEMs for biologically independent replicates (*n* = 5).

A nonsense mutation was created in the *MT-ND5* gene of mice by microinjection of DddA-derived cytosine base editor (DdCBE)-encoded mRNA into 1-cell-stage embryos. This induced the m.C12336T knockout mutation for incorporation of a premature stop codon and m.G12341A bystander edit (silent mutation) in the editing window of *MT-ND5* (Fig. 1a). We intended to achieve mtDNA editing for premature truncation at the 199th amino acid position in the mouse *MT-ND5* gene, where 5′-CAA-3′ glutamine was substituted with a 5′-TAA-3′ premature stop codon with a silent mutation in the 200th glutamine (Fig. 1b). Considering that mtDNA is maternally inherited, we crossed F0 female mice with wild-type (WT) male mice to test germline transmission. We expanded the number of mice harboring the m.C12336T knockout mutation by breeding female mutant mice. The results revealed that 2–48% of the generated mice harbored the m.C12336T knockout mutation (Fig. 1c).

Moreover, these mutations were detected in various tissues of adult mice (F0, mutant-099) at 14 weeks after birth, indicating the induction of a heteroplasmic knockout mtDNA mutation throughout the entire body of each mouse. These results were consistent across the first, second, and third generations of mutant mice (Fig. 1d). These genes were confirmed by Sanger sequencing (Supplementary Fig. 1). Unexpectedly, the genotyping results of individual cells in each organ revealed an extensive heteroplasmy range of the m.C12336T mutation. Furthermore, there was a cell with a 100% homoplasmy mutation rate in brain tissue, and the median heteroplasmy rate of individual cells correlated with that of the whole-organ lysate (Supplementary Fig. 2). To verify the retention of editing frequency over time, we periodically collected fecal samples from mutant mice and genotyped them via targeted deep sequencing. The frequencies of the m.C12336T and m.G12341A mutations remained stable for at least 30 weeks after birth (Fig. 1e).

The primary role of mitochondria is to generate energy by utilizing oxygen^18^. Thus, we assumed that mice with the induced knockout mutation may face challenges in ATP production due to mitochondrial dysfunction. To validate the knockout mutation in the *MT-ND5* gene in mice, we established a mouse embryonic fibroblast (MEF) cell line to assess the oxygen consumption rates of the mutants. Compared with WT MEFs, mutant MEFs presented a significantly lower oxygen consumption rate (Fig. 1f), demonstrating impaired ATP synthesis due to mitochondrial dysfunction in mutant mice.

### Brain damage in *MT-ND5* knockout mice

**Fig. 2:**
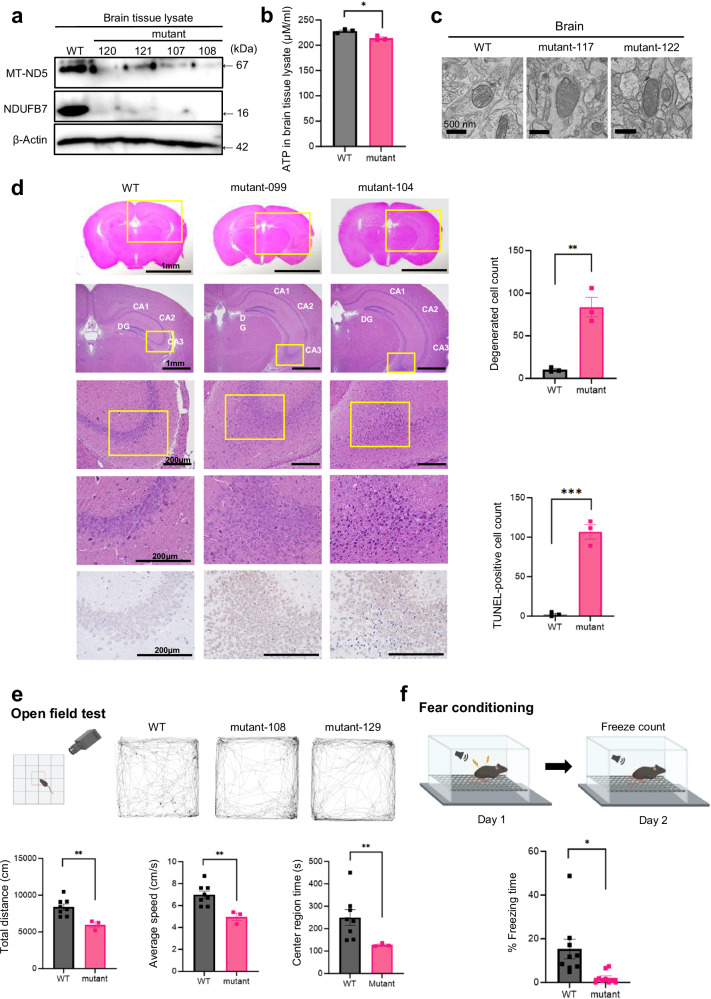
Brain-associated phenotypic analysis of heteroplasmic gene-knockout mutant mice. **a** Protein expression levels of *mt-ND5* and NDUFB7 in WT and mutant mouse brain tissues determined by Western blot analysis. **b** ATP concentrations in the brain tissues of WT and mutant mice. The error bars indicate the SEMs for biologically independent samples (*n* = 3). The exact *p* value was 0.043 (*p < 0.05, **p < 0.01, and ***p < 0.001 according to the Student’s two-tailed *t* test). **c** Transmission electron micrographs of the mitochondria in the brain cerebral cortices of both WT and mutant mice. **d** Histological analysis of H&E- and TUNEL-stained midbrain tissue sections from WT and mutant mice. The enlarged results depict magnifications of the yellow boxed area in the upper rows. Degenerated and TUNEL-positive cells were counted from three randomly selected areas of each sample (200 μm × 200 μm). Error bars indicate the s.e.m. for *n* = 3 independent sections of slides. The exact *p* values are 0.0032 and 0.00030, respectively. (*p < 0.05, **p < 0.01, and ***p < 0.001 via Student’s two-tailed *t* test). DG dentate gyrus, CA cornu ammonis; blue arrowheads: degenerated cells; blue arrows: TUNEL-positive cells. **e** Open field test for the evaluation of memory capacity. The total distance moved (cm), average moving speed (cm/s), and center region dwell time (s) were measured for WT and mutant mice. Error bars indicate the s.e.m. for *n* ≥ 3 biologically independent mice. The exact *p* values were 0.0039, 0.0051, and 0.0097, respectively (*p < 0.05, **p < 0.01, and ***p < 0.001 via two-tailed Student’s *t* test). **f** Fear conditioning test to assess the ability to memorize fear. The graph shows the percentage of freezing time between the two stimulation conditions. The error bars indicate the SEMs for *n* ≥ 3 biologically independent mice. The exact *p* value was 0.011 (*p < 0.05, **p < 0.01, and ***p < 0.001 according to Student’s two-tailed *t* test).

Next, we aimed to investigate whether there was a loss-of-function mutation in the mtDNA necessary for the function of specific organs, particularly those with high oxygen consumption, such as the brain. Despite constituting only ~2% of body weight, the brain consumes >20% of the body’s energy needs in humans^19^. Organs with such high energy consumption are highly dependent on the proper functioning of mitochondria^20^. To determine the protein expression levels of MT-ND5 and complex I in the brains of mutant mice, we conducted Western blot analyses for MT-ND5 and NDUFB7, which are known to be representative proteins identified via quantitative proteomic analysis of complex I^21^. The protein levels of MT-ND5 and NDUFB7 were lower in all the mutant mice than in the WT mice (Fig. 2a). ATP levels in tissue lysates were also lower in mutant mice than in WT mice (Fig. 2b). Additionally, TEM revealed damaged mitochondrial cristae in the cerebral cortex (Fig. 2c). To explore histopathological distinctions, we examined brain tissue sections from both WT and mutant mice via hematoxylin and eosin (H&E) staining. There was no significant difference in the forebrain between WT and mutant mice (Supplementary Fig. 3). However, mutant mice presented asymmetry in the hippocampus within midbrain regions (Fig. 2d). This asymmetry was observed more clearly via IHC with anti-NeuN (Supplementary Fig. 4). We observed a 1.6–2.4-fold difference in size between the left and right hippocampi of mutant mice at 14 weeks after birth (mutant-099 and mutant-104, respectively). Mice with hippocampal asymmetry showed 2.0–11.8% editing efficiency. Nevertheless, there was no statistically significant correlation between the mutant load and asymmetry ratio (Supplementary Fig. 5). Furthermore, intercell spaces were wider in mutant mice than in WT mice, resulting in a thicker layer in the CA3 region of the abnormal hippocampus. Additionally, we observed that the number of apoptotic cells was greater in the hippocampi of mutant mice than in those of WT mice via the TUNEL assay. We performed additional experiments with other apoptotic markers to detect apoptotic responses. Compared with WT mice, mutant mice presented greater expression of cleaved caspase-3 (which is a general apoptotic marker) (Fig. 2d and Supplementary Fig. 4). Following these results, we confirmed the occurrence of apoptosis in neuronal cells within the CA3 region of the hippocampus in mutant mice.

The hippocampus plays a role in short-term memory, learning ability, and behavioral activity by transmitting signals to other brain regions^22,23^. To investigate ethological changes in mutant mice, WT and mutant mice were subjected to an open-field test and fear conditioning. The results showed that mutant mice tended to be hypoactive, demonstrating a 1.4-fold shorter total distance traveled and a 1.4-fold slower movement compared with WT mice. There was also a 2.0-fold reduction in the center region dwell time, indicating that mutant mice had higher anxiety levels than WT mice. Additionally, we performed a fear conditioning test for aversive learning by measuring freezing time by providing shocks and sound stimulation over 24-hour intervals. Only sound stimulation was provided in the second stimulation. Compared with WT mice, heteroplasmic knockout mice presented a shorter freezing time, suggesting a fear-memorization disorder (Fig. 2e, f). Mice with abnormal behavior showed 2.0–21.1% editing efficiency. However, a statistically significant correlation between the mutant load and ethological changes was not observed (Supplementary Fig. 6).

### Fat accumulation in mutant mice

**Fig. 3:**
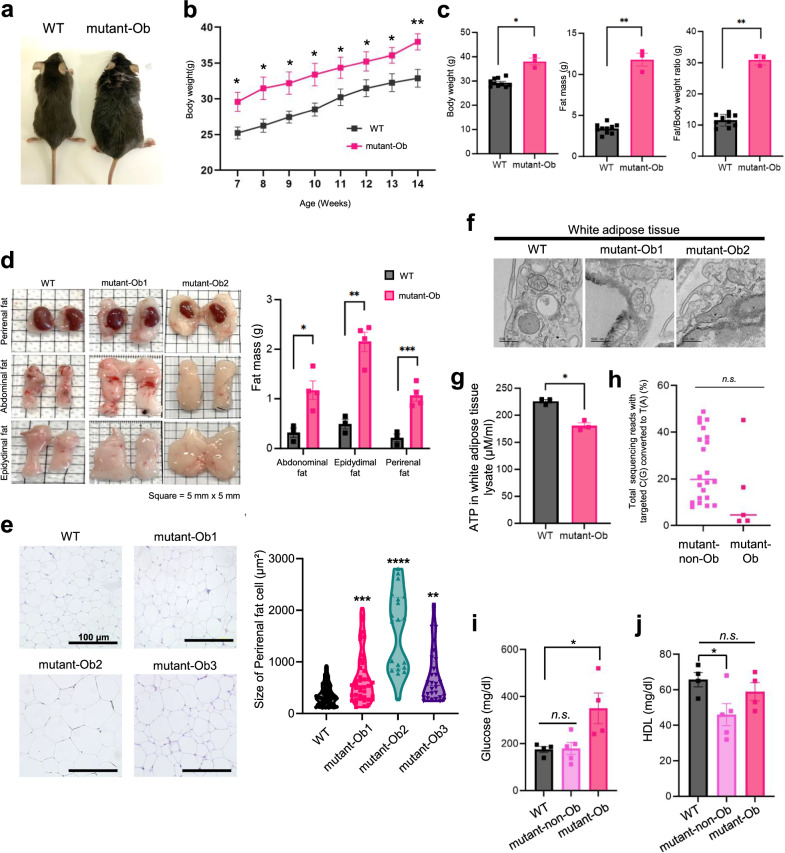
Obesity-phenotype assessments of heteroplasmic gene-knockout mutant mice. **a** Representative images of obese mutant mice. **b** Average body weights of WT (*n* = 5) and obese mutant mice (*n* = 4). The error bars indicate the SEMs for *n* ≥ 4 biologically independent mice. The exact *p* values for 7, 8, 9, 10, 11, 12, 13, and 14 weeks were 0.036, 0.034, 0.046, 0.043, 0.069, 0.077, 0.054, and 0.020, respectively (*p < 0.05, **p < 0.01, and ***p < 0.001 according to a two-tailed Student’s *t* test). **c** Mean body weight, fat mass, and fat/body weight ratio of WT mice (*n* = 10) and mutant-Ob mice (*n* = 3) were measured via dual-energy X-ray absorptiometry. The error bars represent the SEMs for *n* = 3 biologically independent mice. The exact *p* values were 0.015, 0.0058, and 0.00011 (*p < 0.05, **p < 0.01, and ***p < 0.001 according to a two-tailed Student’s *t* test). **d** Images of perirenal fat, abdominal fat, and epidydimal fat tissues from WT and mutant-Ob mice. Each grid in the image is 5 mm long. The measured weights of the fat tissues are shown on the right. The error bars represent the SEMs for *n* = 3 biologically independent mice. The exact *p* values were 0.0018, 0.0013, and 0.0013 for abdominal fat, perirenal fat, and epidydimal fat, respectively (*p < 0.05, **p < 0.01, and ***p < 0.001 via two-tailed Student’s *t* test). **e** (Left) Microscopy image of perirenal fat stained with H&E at ×200 magnification. (Right) Measurement of the diameter of perirenal fat cells. Lines are the means with s.e.m. Exact *p* values were 0.00016, 9.5E-7, and 0.0016 for mutant-Ob1, mutant-Ob2, and mutant-Ob3 mice, respectively (*p < 0.05, **p < 0.01, ***p < 0.001, and ****p < 0.0001, respectively, according to a two-tailed Student’s *t* test). **f** Transmission electron micrographs of mitochondria in white adipose tissues of WT and obese *MT-ND5* gene-knockdown mutant mice. **g** Analysis of ATP synthesis in white adipose tissues from WT (*n* = 3) and mutant (*n* = 3) mice. The error bars indicate the SEMs for biologically independent samples (*n* = 3). The exact *p* value was 0.0021 (*p < 0.05, **p < 0.01, and ***p < 0.001 via two-tailed Student’s *t* test). **h** Efficiency of the distribution of nonobese and obese mutants in mutant mice. The exact *p* value was 0.33 (*n.s*., not significant). **i** Glucose levels in WT (*n* = 4), mutant-non-Ob (*n* = 5), and mutant-Ob (*n* = 4) mice. The error bars indicate the SEMs for biologically independent samples (*n* ≥ 4). The exact *p* values were 0.89 and 0.039 for the mutant-non-Ob and mutant-Ob mice, respectively (*p < 0.05, using Student’s two-tailed *t* test). **j** Measurements of HDL in WT (*n* = 4), mutant-non-Ob (*n* = 5), and mutant-Ob (*n* = 4) mice. The error bars indicate the SEMs for biologically independent samples (*n* ≥ 4). The exact *p* values were 0.035 and 0.340 for the mutant-non-Ob and mutant-Ob mice, respectively (*p < 0.05, using Student’s two-tailed *t* test). HDL: high-density lipoproteins.

Mitochondrial dysfunction resulting from mutations in mtDNA can lead to various metabolic disorders, such as maternally inherited diabetes and deafness (MIDD)^24,25^. The body weights of mutant mice with an obese phenotype (mutant-Ob) were 1.2 times greater than those of WT mice (Fig. 3a, b), whereas not all mutant mice with the knockout genotype presented differences in body weight. Five (29%) of the 17 mutant mice with an obesity-related phenotype presented an increase in body weight from 7 weeks after birth (Table 1). Encouraged by this result, we measured the body compositions of mutant mice via dual-energy X-ray absorptiometry to assess the higher body weights observed in several mutant mice. Consequently, the average body weights and fat masses of the mutant-Ob mice were 1.3- and 3.5-fold greater than those of the WT mice, respectively. The average body fat ratio (fat mass over body weight) of the mutant-Ob mice was 20% greater than that of the WT mice (Fig. 3c). We also conducted an autopsy of three different fat tissues from mutant-Ob mice to examine the abdominal, epididymal, and perirenal fat. These tissues exhibited visible differences. Moreover, the weight of each fat tissue sample was significantly greater than that of the WT mouse tissue, with 3.7-, 4.4-, and 5.0-fold increases in abdominal, epididymal, and perirenal fat, respectively (Fig. 3d). Furthermore, the sizes of the perirenal adipose cells in the mutant-Ob mice were measured via H&E-stained tissue sections, which revealed that the degree of hypertrophy ranged between 1.9 and 4.4 times greater than that in the WT mice (Fig. 3e). Furthermore, TEM images revealed damaged mitochondrial cristae in white adipose tissue (Fig. 3f).

**Table 1:** Summary of the numbers of mutant-Ob and mutant-non-Ob.

The numbers of mutants with fat-related phenotype (*n* = 17)		
Appearance	Obese	Non-obese
m.C12336T-editing efficiency (%)	2–45	3–46
Number of mice (%)	5 (29.4)	12 (70.6)

We also analyzed the levels of biochemical markers in the serum and ATP in white adipose tissue. While other serum markers were not significantly different between the mutant-Ob and WT mice (Supplementary Fig. 7), the glucose levels in the mutant-Ob mice were twice those in the WT mice. Additionally, ATP levels were decreased in mutant-Ob mice (Fig. 3g). Nevertheless, no difference was observed in the editing frequency of the m.C12336T mutation between mutant-Ob mice and those without obesity onset (Fig. 3h). We assumed that *MT-ND5* knockout mutant mice susceptible to obesity may be at risk of metabolic disorders. Thus, we examined the serum levels in WT, mutant-Ob, and mutant-non-Ob mice. In the serum analysis, unlike in the mutant-Ob mice, blood glucose did not increase in the mutant-non-Ob mice, although a decrease in HDL was observed (Fig. 3i, j). HDL, considered instructive cholesterol, has been reported to be low in diabetes patients^26^. However, there were no significant differences in the levels of cholesterol, LDL, triglycerides, phosphor lipids, or phosphorous between the groups (Supplementary Fig. 7). These results suggest that the phenotypic expression and potential induction of obesity are attributed to heteroplasmic knockout mutations.

### *MT-ND5* nonsense mutants are susceptible to obesity

**Fig. 4:**
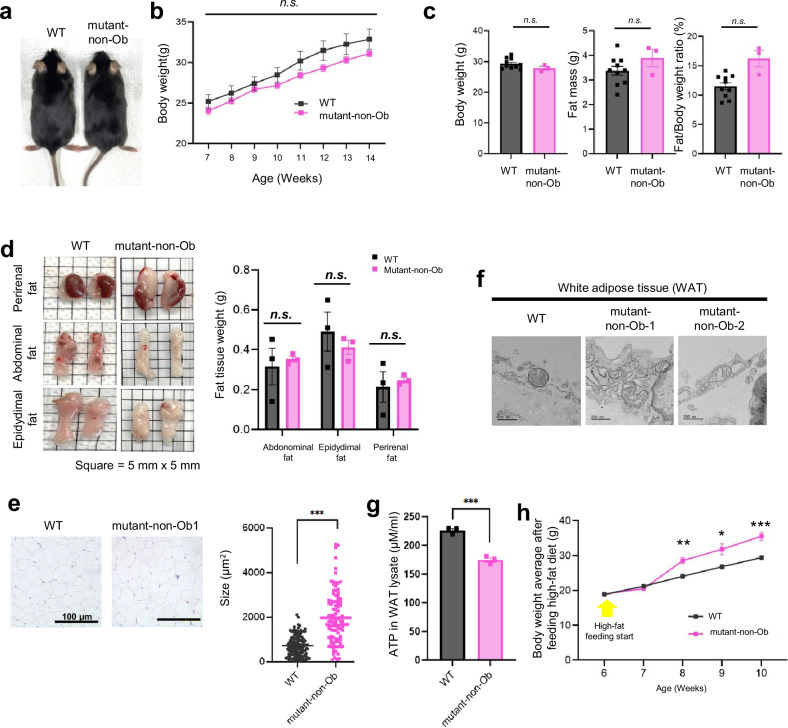
Nonobese phenotype assessments of heteroplasmic gene-knockout mutant mice. **a** Representative images of nonobese mutant mice. **b** Average body weights of WT (*n* = 5) and obese mutant mice (*n* = 10). Error bars indicate the s.e.m. for biologically independent mice (*n* ≥ 5). The exact *p* values for 7, 8, 9, 10, 11, 12, 13, and 14 weeks were 0.27, 0.37, 0.44, 0.23, 0.21, 0.15, 0.21, and 0.23, respectively (*n.s*., not significant, using Student’s two-tailed *t* test). **c** Mean body weight, fat mass, and fat/body weight ratio in WT mice (*n* = 3) and mutant-non-Ob mice (*n* = 3) measured by dual-energy X-ray absorptiometry. The error bars indicate the s.e.m. for biologically independent mice (*n* = 3). The exact *p* values of body weight, fat mass, and the fat mass/body weight ratio were 0.13, 0.28, and 0.057, respectively (*n.s*., not significant; Student’s two-tailed *t* test was used). **d** Images of perirenal fat, abdominal fat, and epidydimal fat tissues from WT and Mutant-non-Ob mice. Each grid in the image is 5 mm long. The measured weights of the fat tissues are shown on the right. The error bars indicate the s.e.m. for biologically independent mice (*n* = 3). The exact *p* values were 0.71, 0.49, and 0.68 for abdominal fat, epidydimal fat, and perirenal fat, respectively (n.s., not significant; Student’s two-tailed *t* test was used). **e** (Left) Microscopy image of perirenal fat via H&E at ×200 magnification. (Right) Measurements of the sizes of perirenal fat cells. Lines represent the mean with s.e.m. The exact *p* value was 2.0E-18 (***p < 0.001 using Student’s two-tailed *t* test). **f** Transmission electron micrographs of mitochondria in white adipose tissues of WT and nonobese mutant mice. **g** Analysis of ATP synthesis in white adipose tissues of WT (*n* = 3) and mutant mice (*n* = 4). The error bars indicate the SEMs for biologically independent samples (*n* ≥ 3). The exact *p* value was 0.0002 (***p < 0.001 using Student’s two-tailed *t* test). **h** Average body weights of WT (*n* = 5) and non-Ob-phenotype mutant mice (*n* = 4) after high-fat feeding (arrow). Error bars indicate the s.e.m. for biologically independent mice (*n* ≥ 3). The exact *p* values for 6, 7, 8, 9, and 10 weeks were 0.77, 0.14, 0.0061, 0.041, and 0.0069, respectively (*p < 0.05, **p < 0.01, and ***p < 0.001 according to a two-tailed Student’s *t* test).

Encouraged by these results, we aimed to investigate differences between mutant-Ob and mutant mice not showing an obese phenotype (mutant-non-Ob). Unlike mutant-Ob mice, mutant-non-Ob mice and WT mice presented no differences in external features, body weight (Fig. 4a, b), body composition (Fig. 4c), or visible distinctions in fat tissue (Fig. 4d). However, several similarities were observed between the mutant-Ob and mutant-non-Ob mice. Similar to those in the mutant-Ob phenotypes, the sizes of white adipose cells in the mutant-non-Ob mice were twice as large as those in the WT mice (Fig. 4e). Furthermore, damaged mitochondrial cristae were observed in white adipose tissues from the mutant-non-Ob comparison (Fig. 4f). Additionally, ATP levels were similarly reduced in white adipose tissue lysates from mutant-non-Ob mice (Fig. 4g). To assess the susceptibility to obesity, we fed a high-fat diet to mutant mice that did not exhibit the obese phenotype with MT-ND5 gene-knockdown mutations. Two weeks after the high-fat diet, the mutant mice exhibited significantly greater weight gain than the WT mice (Fig. 4h). These results suggest that, regardless of the external features of obesity, mitochondrial heteroplasmic knockout mice are highly susceptible to obesity.

### *MT-ND5* nonsense mutation leads to failure of heat production and decreased thermoregulation ability

**Fig. 5:**
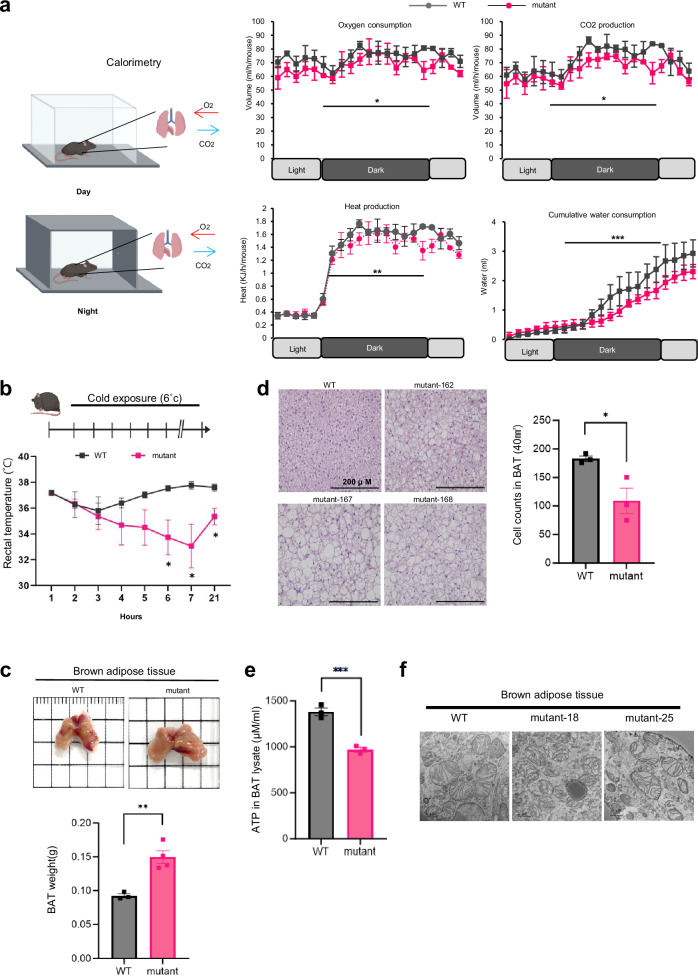
Thermogenesis-associated phenotype analysis in heteroplasmic gene-knockout mutant mice. **a** The results of the calorimetry test for both WT (wild-type) mice (*n* = 3) and mutant mice (*n* = 3). The analysis was based on a time course that included both a light cycle (5 h, 4 h) and a dark cycle (12 h). The results are presented as averages of individual mice. The measurement subsections included oxygen consumption, CO_2_ production, heat production, and cumulative water consumption, as annotated. The error bars indicate the s.e.m. for biologically independent mice (*n* = 3). The exact *p* values were 0.045, 0.041, 0.0084, and 0.00015, respectively (*p < 0.05, **p < 0.01, and ***p < 0.001 according to a two-tailed Student’s *t* test). **b** Average rectal temperatures of WT (*n* = 3) and mutant mice (*n* = 5) after cold challenge. Measurements were made at a temperature of 6 °C with intervals of 1 h. The error bars indicate the SEMs for biologically independent samples (*n* ≥ 3). The exact *p* values were 0.60, 0.95, 0.78, 0.33, 0.14, 0.047, 0.049, and 0.033 for 0, 1, 2, 3, 4, 5, 6, and 21 hours, respectively (*p < 0.05 via a two-tailed Student’s *t* test). **c** Images of brown adipose tissues from WT (*n* = 3) and mutant mice (*n* = 4) with corresponding bar graphs. Each grid in the image is 5 mm long. The error bars indicate the SEMs for biologically independent samples (*n* ≥ 3). The exact *p* value was 0.0065 (**p < 0.01 according to a two-tailed Student’s *t* test). **d** Histological analysis of brown adipose tissue sections from WT and mutant mice via H&E staining. Measurements of brown adipocyte counts are presented on the right. The error bars indicate the SEMs for biologically independent samples (*n* ≥ 3). The exact *p* value was 0.030 (*p < 0.05 using the Student’s two-tailed *t* test). **e** Analysis of ATP synthesis in brown adipose tissues from WT (*n* = 3) and mutant (*n* = 3) mice. The error bars indicate the SEMs for biologically independent samples (*n* = 3). The exact *p* value was 0.0001 (*p < 0.05, **p < 0.01, and ***p < 0.001 according to the Student’s two-tailed *t* test). **f** Transmission electron micrographs of the mitochondria in brown adipose tissues of WT and mutant mice.

One of the most essential functions of mitochondria is thermoregulation, where brown adipose tissue (BAT), a mitochondria-rich tissue, modulates thermogenesis^27^. We hypothesized that mitochondrial heteroplasmic gene knockout could induce thermogenic disorders. Thus, we conducted an indirect calorimetry test to assess the metabolic abilities of mutant mice. As shown in Fig. 5a, the oxygen consumption and CO_2_ production rates of the mutant mice were reduced during the dark cycle. Decreased metabolic ability led to the failure of heat production and abnormal drinking behavior (Fig. 5a). Assuming that mutant mice have decreased heat production ability, we exposed both WT and mutant mice to a cold environment (6°C) to confirm abnormal thermogenesis in the mutant mice. We observed a gradual decrease in rectal temperature over several hours. However, there were significant temperature decreases in the mutant mice after 5 hours. Furthermore, one mutant mouse died after 21 hours, and the remaining mutant mice also presented decreased rectal temperatures compared with those of WT mice (Fig. 5b). After the cold challenge, BATs from the mice were collected through autopsy. As observed in white adipose tissues (Fig. 3), BATs of mutant mice appeared larger. These mice were 1.8 times heavier than the WT mice (Fig. 5c). The average cell size of brown adipocytes from mutant mice was also 1.8 times larger than that of brown adipocytes from WT mice (Fig. 5d). Additionally, compared with WT mice, mutant mice presented decreased ATP production in BAT (Fig. 5e). Damaged mitochondrial cristae were also observed in the BATs of the mutant mice (Fig. 5f). Taken together, these results demonstrated that mutant mice failed to produce heat and exhibited impaired thermoregulation ability due to mitochondrial dysfunction induced by the *MT-ND5* gene nonsense mutation.

### Off-target effects in the *MT-ND5* nonsense mutant

A previous study revealed that DdCBE-mediated mtDNA editing could generate off-target activity in nuclear DNA^14,28,29^ and mitochondria^30^. Thus, we conducted whole-mitochondrial genome-wide sequencing in mutant and mutant-Ob mice. The results revealed that mutant mice presented several off-target activities at the entire mtDNA locus with less than 5% editing efficiency, and mutant-Ob mice presented only a few instances of off-target editing (Supplementary Fig. 8). Furthermore, we chose off-target sites harboring a perfect match or single nucleotide mismatch with the left or right TALE binding sequence in the nuclear genome (Supplementary Table 2). Compared with WT mice, mutant and mutant-Ob mice, whose editing efficiency of nonsense mutations ranged from 1% to 17%, presented negligible off-target editing frequencies (Supplementary Fig. 9). These results suggest that off-target editing has no apparent relationship with phenotypic expression in mitochondrial knockout mutants.

## Discussion

In this study, we elucidated diverse phenotypes associated with the mitochondrial gene *MT-ND5* knockout mutation induced by the incorporation of a premature stop codon at the 199th position in the target gene. The heteroplasmic knockout, presumed to be a loss-of-function mutation, was successfully achieved, with an editing frequency of up to 48%, and the desired mutations were observed in various tissues. Our findings demonstrate that microinjection of DdCBE-encoding mRNA followed by consecutive crossings can yield sufficient *MT-ND5* gene-knockdown mice for phenotype analysis without causing mtDNA copy number loss.

Notably, these mice presented diverse editing frequencies of the m.C12336T mutation, along with phenotypes related to metabolism and the brain. Mitochondrial dysfunction is recognized as the epicenter of energy metabolism. It has been implicated in the pathophysiology of a variety of metabolic and neurodegenerative diseases that can affect vital organs, including the brain, heart, muscles, liver, eyes, and pancreas^31^. In particular, mitochondria in the brain are essential for important biological processes, as they are involved in various biological functions, such as energy and free radical production, cell metabolism, neurotransmitter regulation, apoptosis, and inflammation in the brain^31–33^. Recent studies have highlighted mitochondrial dysfunction as a significant contributor to brain disorders, with hippocampal asymmetry being a notable feature in conditions such as Alzheimer’s disease and Parkinson’s disease^34^. We observed a simultaneous decrease in the protein expression of MT-ND5 and complex I, as well as diminished ATP levels in the brains of mutant mice. Additionally, severe damage to the cristae of mitochondria was detected. Mitochondria serve as primary sites for ATP production, where mitochondrial respiration chains, especially complex I, are involved in this process^35^. Furthermore, through histopathological analysis, abnormalities in the hippocampus, including asymmetrical hippocampal atrophy and an increase in degenerated and apoptotic cells in CA3, were observed. Consequently, abnormalities in learning memory were confirmed through behavioral assessment. Ethological changes can occur in mutant mice because the hippocampus plays a role in short-term memory, learning ability, and behavioral activity by transmitting signals to other parts of the brain^22,23^. Specifically, intercell spaces were wider in mutant mice than in WT mice. The number of condensed nuclei and apoptotic cells was greater in the hippocampi of mutant mice than in those of WT mice. Given the crucial role of the CA3 region in encoding spatial representations and episodic memories, these results suggest that a nonsense mutation in the mitochondrial gene can lead to structural abnormalities in the brain with corresponding behavioral differences. This model could serve as a valuable method for studying therapies for mitochondria-related brain diseases.

Mitochondrial dysfunction may lead to the pathogenesis of metabolic disorders in tissues involved in nutrient metabolism^36^. In particular, mitochondria in white adipose tissues are the main source of ATP and play critical roles in adipocyte biological processes such as adipogenesis, lipolysis, and fatty acid oxidation^37,38^. We observed that 30% of the mutant mice exhibited a natural increase in body weight by 1.2-fold. These mice presented severely damaged mitochondria in white adipose tissue, significantly reduced ATP levels, and substantial increases in body fat mass and blood glucose. Furthermore, in mutant-non-Ob mice, which do not naturally gain weight, we observed severely damaged mitochondrial cristae, increased adipocyte size, and significantly reduced ATP and HDL levels. When these mutant-non-Ob mice were fed a high-fat diet, they appeared to gain weight more rapidly than WT mice. These results demonstrate that MT-ND5 knockout can closely influence the metabolic obesity phenotype. Moreover, previous studies have suggested that mitochondria in adipocytes play a role in regulating insulin sensitivity^39^. BrAT e specializes in consuming energy through heat production to maintain body temperature homeostasis^40^. We observed damaged mitochondrial cristae in mutant BATs and a significant decrease in ATP levels. Moreover, compared with WT mice, mutant mice had difficulty maintaining body temperature. Using our animal models, we demonstrated that mitochondria are essential organelles for maintaining the function of adipocytes in metabolic homeostasis, providing useful tools for research on mitochondrial metabolic ability and disease.

In this study, we conducted a comprehensive analysis of the phenotypes of heteroplasmic MT-ND5 gene-knockout mice, exploring the therapeutic potential of MT-ND5 for human mitochondrial disorders. Initially, we hypothesized a correlation between the knockout genotype and the corresponding phenotype. However, in knockouts with correction efficiencies ranging from 2% to 48%, there was no statistically significant difference between the knockdown mutation load and the extent of phenotypic expression, unlike typical point mutations in the nuclear genome. We posit that mtDNA point mutation accumulation may surpass certain thresholds, eventually leading to phenotypic expression^41–43^. Moreover, the well-known mitochondrial pathogenic mutation m.A3243G has been implicated not only in causing mitochondrial encephalomyopathy with lactic acidosis and stroke-like episodes (MELAS) but also as a factor in MIDD^44–46^. According to recent reports, the reason that the same mutation can cause different disorders lies in heteroplasmy^47,48^. A lower heteroplasmy of m.A3243G in MIDD patients than in MELAS patients has been reported^47^. Similarly, if the efficiency of m.C12336T exceeds 48%, there is also a possibility of another phenotype emerging. Additionally, through single-cell analysis, we discovered that there is a variety of heteroplasmy at the cellular level, which suggests that even with the same editing efficiency, differences in the distribution of single cells within the tissue could lead to variations in the degree of phenotypic expression. Several studies have reported that the average heteroplasmy level of a single cell is determined by the proportion of mtDNA molecules with different heteroplasmy levels^49,50^. In our case, there was no statistical correlation between genotype and phenotype expression in the obesity phenotypes (Figs. 3 and 4). However, the causes of the different phenotypic expression patterns in each individual are unclear. Based on several studies on single-cell mtDNA^49,50^, we assumed that variations in the heteroplasmy level of single-cell mtDNA molecules could determine the expression of the phenotype. The phenotype could be expressed when the heteroplasmy level of mtDNA molecules in a single cell is distributed largely at intermediate levels. However, when the deviation of mtDNA molecule heteroplasmy levels in a single cell is large, the dysfunction of single cells harboring high levels of heteroplasmy mtDNA molecules could contribute to phenotypic expression. This phenomenon should be investigated further and could be a great key for mitochondrial gene therapy.

**Fig. 6:**
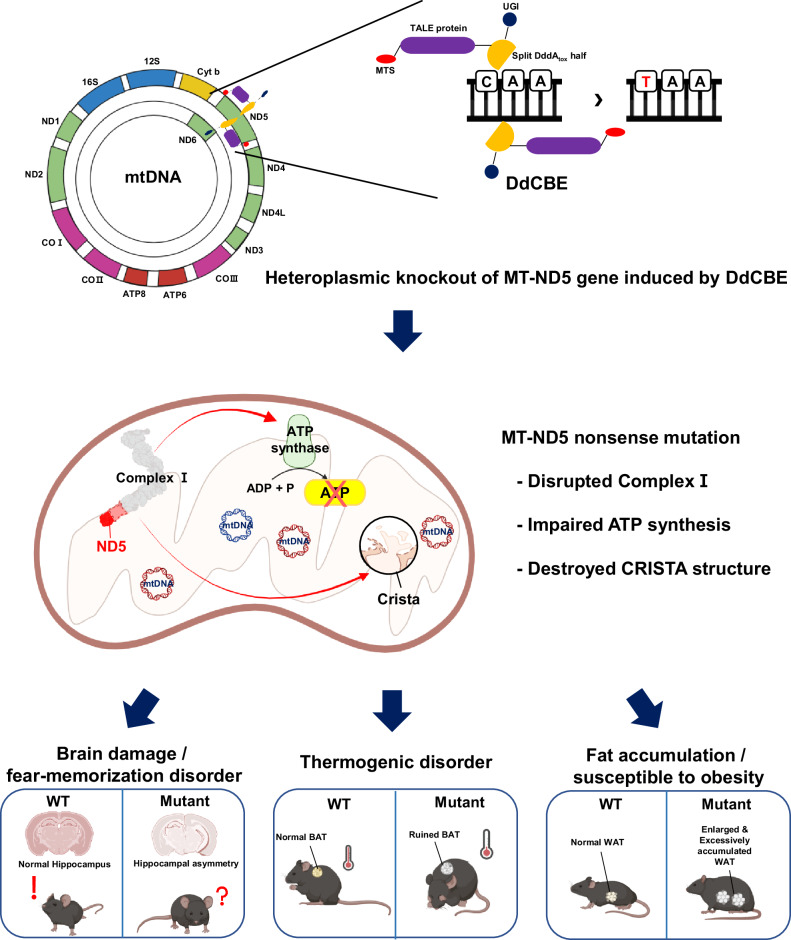
Schematic illustration of the generation of the heteroplasmic MT-ND5 gene knockout and its effects on mitochondria and the resulting phenotype. Heteroplasmic knockout of the MT-ND5 gene induced by DdCBE results in the disruption of Complex I, impaired ATP synthesis, and destruction of the CRISTA structure. These defects lead to brain- and fat-related phenotypes.

In conclusion, our findings contribute to the characterization of an animal model for heteroplasmic MT-ND5 gene-knockout mutation induced by programmable base editing in mtDNA. Our study revealed the harmful effects of mitochondrial gene knockout, which has not been previously reported (Fig. 6). However, investigating specific and contextual mechanisms or pathways related to phenotype expression should be complemented by tissue- or cell-specific knockout systems. Taken together, our results reveal the precise phenotypic features of mitochondrial heteroplasmic knockout, indicating that these mice could serve as valuable animal models for studying mitochondrial dysfunction and developing therapies for human mitochondrial diseases.

## Supplementary information

Supplementary Information

## Data Availability

Supplementary information is available in the online version of the paper. The data that support the findings of this study are available from the corresponding author upon request. The high-throughput sequencing data from this study have been deposited in the NCBI Sequence Read Archive (SRA) database under the accession codes PRJNA1172937. Source data are provided with this paper.
