# Supplementary material for: Comprehensive phenotypic assessment of nonsense mutations in mitochondrial ND5 in mice
Source: Exp Mol Med. 2024 Nov 1;56(11):2395–408. doi: 10.1038/s12276-024-01333-9 (PMC11612467; doi:10.1038/s12276-024-01333-9)
Supplement: Supplementary file 1 — Supplementary Information [file 12276_2024_1333_MOESM1_ESM.pdf]

Supplementary Information

Comprehensive phenotypic assessment of nonsense mutations in mitochondrial ND5 in mice

Contents

Supplementary Table 1. Primers used in this study

Supplementary Table 2. List of potential off-target site

Supplementary Fig. 1. Sanger sequencing chromatogram peaks from various tissues of mutant-107 mice.

Supplementary Fig. 2. Single-cell genotype variation of m.C12336T in each organ.

Supplementary Fig. 3. Histological analysis of wild-type and mutant forebrains.

Supplementary Fig. 4. Immunohistochemistry analysis of wild-type and mutant hippocampus CA3 region

Supplementary Fig. 5. Correlation between editing efficiency and hippocampus asymmetry in mutant mice.

Supplementary Fig. 6. Correlations between editing efficiency and results of behavioral experiments in mutant mice.

Supplementary Fig. 7. Metabolic bio-marker assessments in sera of wild-type ($n = 4$) and mutant mice ($n \geq 3$).

Supplementary Fig. 8. Off-target activity induced by DdCBE in whole mitochondrial genome of wild-type, mutant-non-Ob, and mutant-Ob mice.

Supplementary Fig. 9. Off-target activity induced by DdCBE in nuclear genome of wild-type, mutant-non-Ob, and mutant-Ob mice.

Supplementary Table 1. Primers used in this study

Name	Sequence	Note
mtND5C12336T-1st_S	CACCTCAGCCAACAACAT	mtDNA NGS
mtND5C12336T-1st_AS	TTGGGTGAGAGCACAAATAG	mtDNA NGS
mtND5C12336T-2nd_S	ACACTCTTTCCCTACACGACGCTCTTCCGATCTCCCTACAAGCAATCCTCTATAAC	mtDNA NGS
mtND5C12336T-2nd_AS	GTGACTGGAGTTCAGACGTGTGCTCTTCCGATCTGAGGCCAAATTGTGCTGA	mtDNA NGS
OT1_1st_S	CCATAGCAAGAATGGGACAA	Off-target NGS
OT1_1st_AS	CGCCATAGACCAAGATCAAA	Off-target NGS
OT1_2nd_S	ACACTCTTTCCCTACACGACGCTCTTCCGATCTAGAACAGGGCCAGGTAAA	Off-target NGS
OT1_2nd_AS	GTGACTGGAGTTCAGACGTGTGCTCTTCCGATCTCACACCCTATCCTTCAGTCA	Off-target NGS
OT2_1st_S	ATGCAGGTTAAAGACACTCC	Off-target NGS
OT2_1st_AS	GAATTCACAGGTGGGAAAGA	Off-target NGS
OT2_2nd_S	ACACTCTTTCCCTACACGACGCTCTTCCGATCTGGGAGCACCCTCTAAGATA	Off-target NGS
OT2_2nd_AS	GTGACTGGAGTTCAGACGTGTGCTCTTCCGATCTCTGCATAGCCCTTGTCAC	Off-target NGS
OT3_1st_S	ATACATCTGGGACAACACATC	Off-target NGS
OT3_1st_AS	GCTTGTTCCTCCTGTGATT	Off-target NGS
OT3_2nd_S	ACACTCTTTCCCTACACGACGCTCTTCCGATCTCAGCCCTACAAGCAATTCTA	Off-target NGS
OT3_2nd_AS	GTGACTGGAGTTCAGACGTGTGCTCTTCCGATCTGAGTCTCAGTGGTCAGAGTA	Off-target NGS
OT4_1st_S	CCCTGTGTTCCATCCAATAG	Off-target NGS
OT4_1st_AS	GAAAGTTCCTCCACAGTACC	Off-target NGS
OT4_2nd_S	ACACTCTTTCCCTACACGACGCTCTTCCGATCTGTCTGATCCTAGAATCGCAAATA	Off-target NGS
OT4_2nd_AS	GTGACTGGAGTTCAGACGTGTGCTCTTCCGATCTGGCTGCTCACTGTTTCTT	Off-target NGS
OT5_1st_S	ACTTAGAAATCCAGGGTGAAG	Off-target NGS
OT5_1st_AS	CAGCCTGACTCATCTTACTG	Off-target NGS
OT5_2nd_S	ACACTCTTTCCCTACACGACGCTCTTCCGATCTAGTTTGGGCTTCTGAAAGAT	Off-target NGS
OT5_2nd_AS	GTGACTGGAGTTCAGACGTGTGCTCTTCCGATCTTGAATCCTGACACTTTAGCC	Off-target NGS
OT6_1st_S	TCCATCACAACTAACCCTAATC	Off-target NGS
OT6_1st_AS	GCGTGGCTTTCTCCTTT	Off-target NGS
OT6_2nd_S	ACACTCTTTCCCTACACGACGCTCTTCCGATCTCCTTGCGTTCTCCTACATAAG	Off-target NGS
OT6_2nd_AS	GTGACTGGAGTTCAGACGTGTGCTCTTCCGATCTGTGGAGCAGCGAATTAGAG	Off-target NGS
OT7_1st_S	CTTCCCTATTCAGAGGCTAATC	Off-target NGS
OT7_1st_AS	GGCAATGTGAATGCTATGC	Off-target NGS
OT7_2nd_S	ACACTCTTTCCCTACACGACGCTCTTCCGATCTCTGTCTCACATGCTTCATTAC	Off-target NGS
OT7_2nd_AS	GTGACTGGAGTTCAGACGTGTGCTCTTCCGATCTCACATGGGGAAATGTCTAGA	Off-target NGS
OT8_1st_S	TTGCTTTCCTCTTCTCTCATT	Off-target NGS
OT8_1st_AS	GCCCTCTTTAGTTGTTAGCC	Off-target NGS
OT8_2nd_S	ACACTCTTTCCCTACACGACGCTCTTCCGATCTTAAGCTTGGGACAACAGATAAA	Off-target NGS
OT8_2nd_AS	GTGACTGGAGTTCAGACGTGTGCTCTTCCGATCTATATCACTCTTGCTGAGTATGC	Off-target NGS
OT9_1st_S	CACAGAGGAGTGTGTGTTAG	Off-target NGS
OT9_1st_AS	GCTATGATGGAGGTTGACTATC	Off-target NGS

OT9_2nd_S	ACACTCTTTCCCTACACGACGCTCTTCCGATCTGGTTGCCAAAGACCTACAA	Off-target NGS
OT9_2nd_AS	GTGACTGGAGTTCAGACGTGTGCTCTTCCGATCTTGGAGGGCTGAGTACATT	Off-target NGS
OT10_1st_S	CTCTAAAGCAAACCGCAAAG	Off-target NGS
OT10_1st_AS	GGCCCAACATGGATAAGTAA	Off-target NGS
OT10_2nd_S	ACACTCTTTCCCTACACGACGCTCTTCCGATCTCCAGGTCTCAAGCTTTGTT	Off-target NGS
OT10_2nd_AS	GTGACTGGAGTTCAGACGTGTGCTCTTCCGATCTTGGGAATGGACCTGTTAGTA	Off-target NGS
OT11_1st_S	TAGTCCTACAGAACAGAAGAGG	Off-target NGS
OT11_1st_AS	GAAGCCTGTCATGGAATCAG	Off-target NGS
OT11_2nd_S	ACACTCTTTCCCTACACGACGCTCTTCCGATCTACTTGTGGTTCCTGCATAAT	Off-target NGS
OT11_2nd_AS	GTGACTGGAGTTCAGACGTGTGCTCTTCCGATCTGCCTCGAACTCAGAAATCC	Off-target NGS
OT12_1st_S	GGCTTGCTTAAACTTTGCTAAT	Off-target NGS
OT12_1st_AS	GCTAGAAGGCTACTCCTACTC	Off-target NGS
OT12_2nd_S	ACACTCTTTCCCTACACGACGCTCTTCCGATCTGAGTTTCAAGAAGAATACATAGCC	Off-target NGS
OT12_2nd_AS	GTGACTGGAGTTCAGACGTGTGCTCTTCCGATCTGCAAAGCTCCTACTACCTAAG	Off-target NGS
OT13_1st_S	AACATATATAGGCACGCCTTC	Off-target NGS
OT13_1st_AS	GCCTAAGTTGGTCTCATAAGG	Off-target NGS
OT13_2nd_S	ACACTCTTTCCCTACACGACGCTCTTCCGATCTCCAACTTCAGTGCAACATA	Off-target NGS
OT13_2nd_AS	GTGACTGGAGTTCAGACGTGTGCTCTTCCGATCTGCTAGACAATGTCATATGCT	Off-target NGS
OT14_1st_S	AGACAAATCTCCCAGCAAAG	Off-target NGS
OT14_1st_AS	ACCTGGCTGACCATGATA	Off-target NGS
OT14_2nd_S	ACACTCTTTCCCTACACGACGCTCTTCCGATCTTCCATGATGAGGAGTGAAGA	Off-target NGS
OT14_2nd_AS	GTGACTGGAGTTCAGACGTGTGCTCTTCCGATCTGACCTCAACAAGAGGGAAAC	Off-target NGS
OT15_1st_S	GAGTTCAGACAACAGGAGTG	Off-target NGS
OT15_1st_AS	ACATAAGGAGACTTGGGTTTG	Off-target NGS
OT15_2nd_S	ACACTCTTTCCCTACACGACGCTCTTCCGATCTCCTGAAATTGAGATGGGCTATAA	Off-target NGS
OT15_2nd_AS	GTGACTGGAGTTCAGACGTGTGCTCTTCCGATCTCCTCTCTCTTCTGGTTTGAATG	Off-target NGS
OT16_1st_S	GGTCCACATTCGGGTATTT	Off-target NGS
OT16_1st_AS	GTGTGTGTGTGTGTTGGA	Off-target NGS
OT16_2nd_S	ACACTCTTTCCCTACACGACGCTCTTCCGATCTGCTTCCTAGCCAACAACTT	Off-target NGS
OT16_2nd_AS	GTGACTGGAGTTCAGACGTGTGCTCTTCCGATCTAGCATTGCCAAAGATAGGG	Off-target NGS
OT17_1st_S	TGCCTTCTGCTCTCTTCT	Off-target NGS
OT17_1st_AS	CACACACACACACCGTAAT	Off-target NGS
OT17_2nd_S	ACACTCTTTCCCTACACGACGCTCTTCCGATCTTGCCCAAGAAATGTAAGGAA	Off-target NGS
OT17_2nd_AS	GTGACTGGAGTTCAGACGTGTGCTCTTCCGATCTTGAGTCCAGGGTATCATTCT	Off-target NGS
OT18_1st_S	GGTGATATGGAGACCCTACT	Off-target NGS
OT18_1st_AS	CTGTAGCTCATTGCCATCAT	Off-target NGS
OT18_2nd_S	ACACTCTTTCCCTACACGACGCTCTTCCGATCTGAGCCATCCTAACACACAC	Off-target NGS
OT18_2nd_AS	GTGACTGGAGTTCAGACGTGTGCTCTTCCGATCTGCTAAGCCTCTCTCTGTTTG	Off-target NGS
OT19_1st_S	TGATGATCACCGTTTCAATACT	Off-target NGS
OT19_1st_AS	TTCGCCTCAATTTCCAATAAAC	Off-target NGS

OT19_2nd_S	ACACTCTTTCCCTACACGACGCTCTTCCGATCTCTGTCATTGTATTATGTAACTACCC	Off-target NGS
OT19_2nd_AS	GTGACTGGAGTTCAGACGTGTGCTCTTCCGATCTATGCAAACCACATTGTCTTC	Off-target NGS
OT20_1st_S	AGGTGACCCTCTGTGAAA	Off-target NGS
OT20_1st_AS	CCACACACCTCACAAAGTAA	Off-target NGS
OT20_2nd_S	ACACTCTTTCCCTACACGACGCTCTTCCGATCTGACAACCAGCTCACTCAAT	Off-target NGS
OT20_2nd_AS	GTGACTGGAGTTCAGACGTGTGCTCTTCCGATCTCACCATGACCTCTGTTCTTT	Off-target NGS
OT21_1st_S	TGTTGCACTGGGTGATTC	Off-target NGS
OT21_1st_AS	GTCATGTGGCTTTCATCCT	Off-target NGS
OT21_2nd_S	ACACTCTTTCCCTACACGACGCTCTTCCGATCTGCTGATGAGCCATACACATTA	Off-target NGS
OT21_2nd_AS	GTGACTGGAGTTCAGACGTGTGCTCTTCCGATCTCTGTGGACTCTTACATCAAGAATA	Off-target NGS
OT22_1st_S	GTTGTGAAAGGCACATGAAC	Off-target NGS
OT22_1st_AS	TGTTTAGAGGACTGGTAGGG	Off-target NGS
OT22_2nd_S	ACACTCTTTCCCTACACGACGCTCTTCCGATCTGTTGTGAAAGGCACATGAAC	Off-target NGS
OT22_2nd_AS	GTGACTGGAGTTCAGACGTGTGCTCTTCCGATCTAAATCCCTGTCCTTGTAATCTT	Off-target NGS
OT23_1st_S	GAAGAGTTTCCACACCTCAT	Off-target NGS
OT23_1st_AS	TTCTACCAGAAGACCCATCTA	Off-target NGS
OT23_2nd_S	ACACTCTTTCCCTACACGACGCTCTTCCGATCTGTAAGCAGCTGTAAGGATGAA	Off-target NGS
OT23_2nd_AS	GTGACTGGAGTTCAGACGTGTGCTCTTCCGATCTCACCATACCACAAGGACAC	Off-target NGS

Supplementary Table 2. List of potential off-target sites

Name	TALE	Sequence (5'-3')	Chromosome	Position	Direction	Mismatches
ON-target	Left	TTCCCTAAACATAAACTCAT	ChrM	12307	+	0
OT1	Left	TTCCCTAAACATcAACTCAT	Chr6	17992659	-	1
OT2	Left	TTCCCTAAACtTAAACTCAT	Chr13	46774259	+	1
OT3	Left	TTCCCTAAACATAAACTCAT	Chr18	3639108	+	0
ON-target	Right	TTGTTGTTGGAGAATAT	ChrM	12344	-	0
OT4	Right	TTGTTGTTGGgGAATAT	Chr1	71179576	-	1
OT5	Right	TTGTTGTTGaAGAATAT	Chr2	140346318	+	1
OT6	Right	TTGcTGTTGGAGAATAT	Chr3	100753996	+	1
OT7	Right	TTGTTaTTGGAGAATAT	Chr4	31111849	-	1
OT8	Right	TTGTaGTTGGAGAATAT	Chr5	82826458	+	1
OT9	Right	TTGTTGaTGGAGAATAT	Chr6	46007153	-	1
OT10	Right	TTGTTGTTGGAcAATAT	Chr7	64940236	+	1
OT11	Right	TTGTTGTTGGAGAATAa	Chr8	83878545	-	1
OT12	Right	TTGTTtTTGGAGAATAT	Chr9	103373850	+	1
OT13	Right	TTGTTGTTGGAGAATAT	Chr10	109221588	-	0
OT14	Right	TTGTTGTTGGAGAATAT	Chr11	59281819	+	0
OT15	Right	TTGTTGTgGGAGAATAT	Chr12	10829818	-	1
OT16	Right	TTGTTGTTGGAaAATAT	Chr13	47851071	+	1
OT17	Right	TTGTTGTTtGAGAATAT	Chr14	45908965	+	1
OT18	Right	TTGTTGTTGGAGAATtT	Chr15	79647715	+	1
OT19	Right	TTGTTGTTGGAGAAaAT	Chr16	61529810	-	1
OT20	Right	TTGTTGTTGGAGAtTAT	Chr17	10268410	+	1
OT21	Right	TTGTTGTTGGAGAcTAT	Chr18	86739046	+	1
OT22	Right	TTGTTGTTGGAGtATAT	Chr19	49787015	-	1
OT23	Right	TTGTTGTTGaAGAATAT	Chrx	107381360	+	1

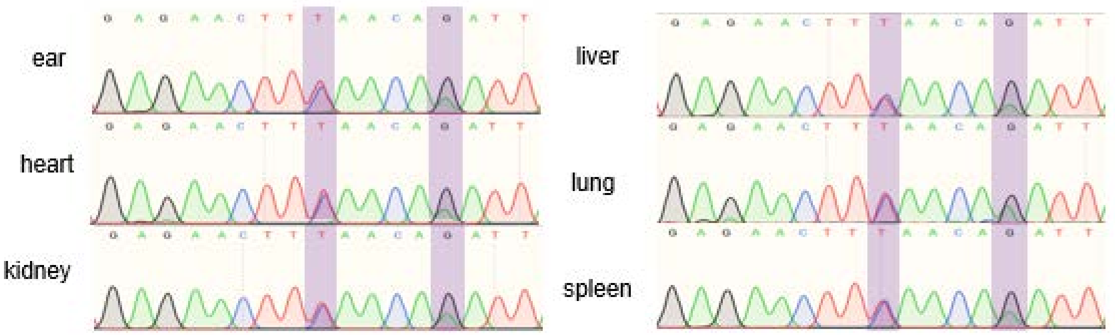

Supplementary Fig. 1. Sanger sequencing chromatogram peaks from various tissues of mutant-107 mice. Base editing targets are m. C12336T (first) and m.G12341A (second) in purple highlight.

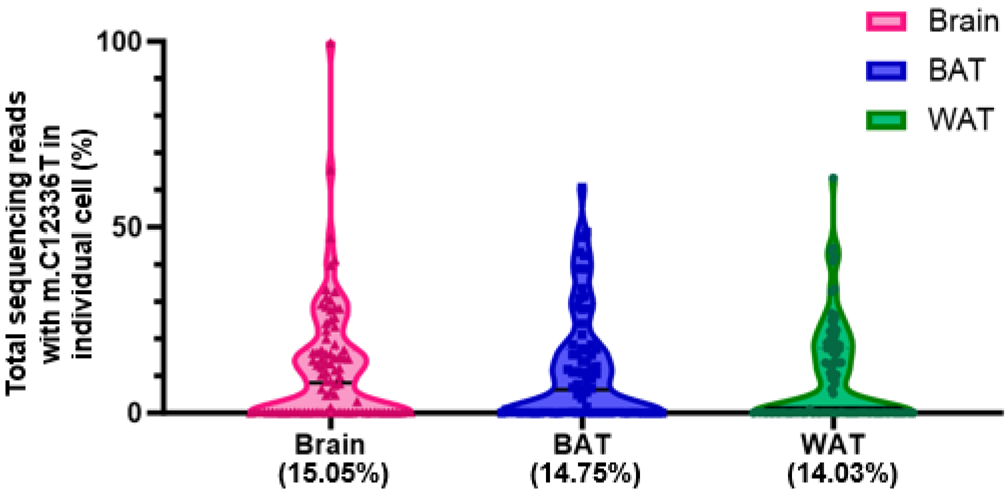

Supplementary Fig. 2. Single-cell genotype variation of m.C12336T in each organ. Editing efficiency of m.C12336T in individual cell from each organ of mutant mice. The number of individual cells were 88, 78, and 92 in brain, BAT, and WAT. Dots mean the editing efficiency of m.C12336T in each individual cells, and the parentheses on the x-axis indicate editing efficiency of m.C12336T in whole organ lysate.

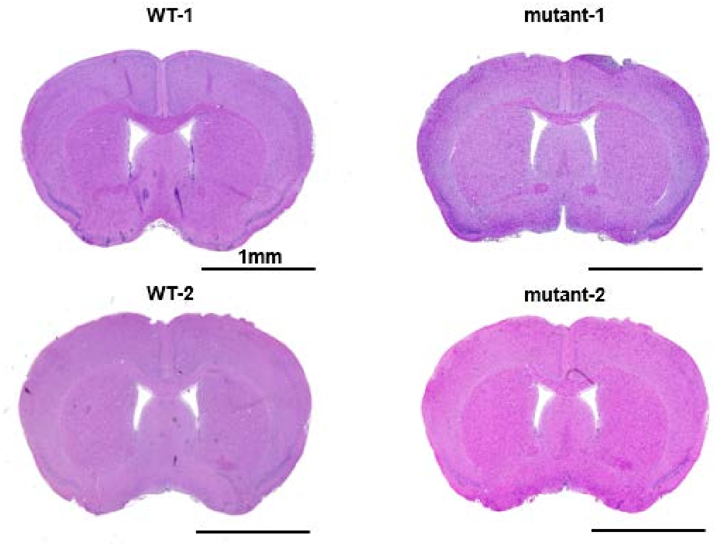

Supplementary Fig. 3. Histological analysis of wild-type and mutant forebrains. Microscopic images of forebrain stained using hematoxylin and eosin staining at 20X magnification.

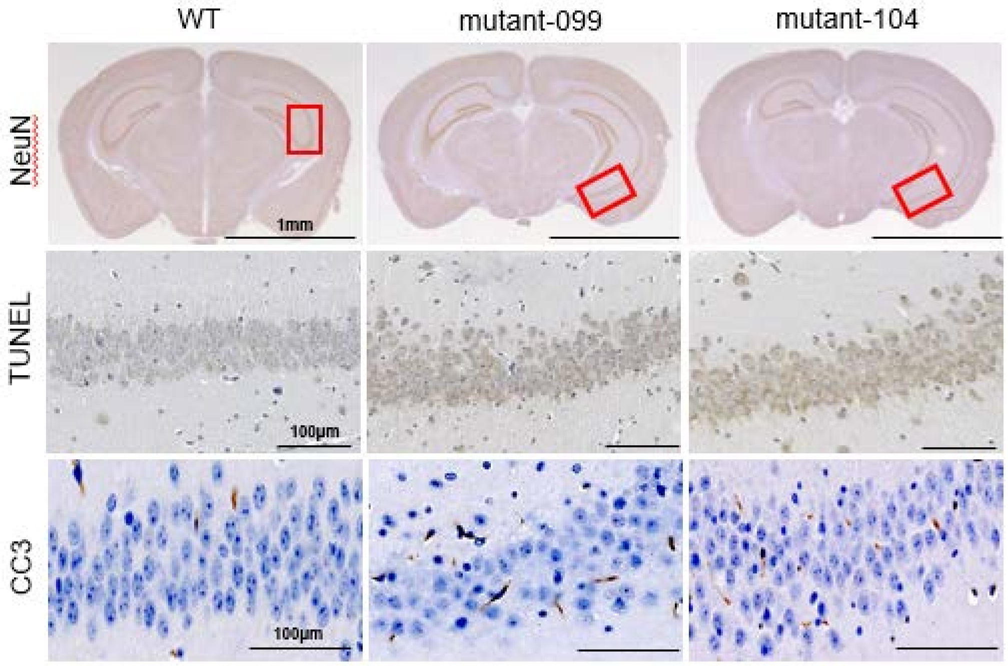

Supplementary Fig. 4. Immunohistochemistry analysis of wild-type and mutant hippocampal CA3 region

Immunohistochemistry staining of neuronal (NeuN), and apoptotic markers (TUNEL, Cleaved Caspase-3) in wild-type and mutant midbrain hippocampus CA3 region.

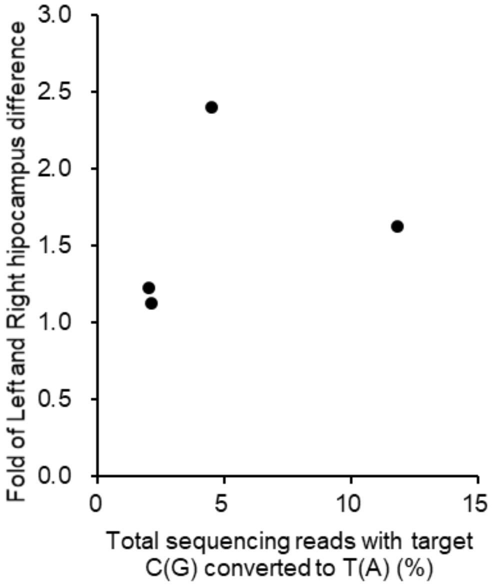

Supplementary Fig. 5. Correlation between editing efficiency and hippocampus asymmetry in mutant mice. This graph shows the time of size difference in the right and left hippocampus (y-axis) and m.C12336T editing efficiency (x-axis) of mice with histological analysis of the hippocampus performed.

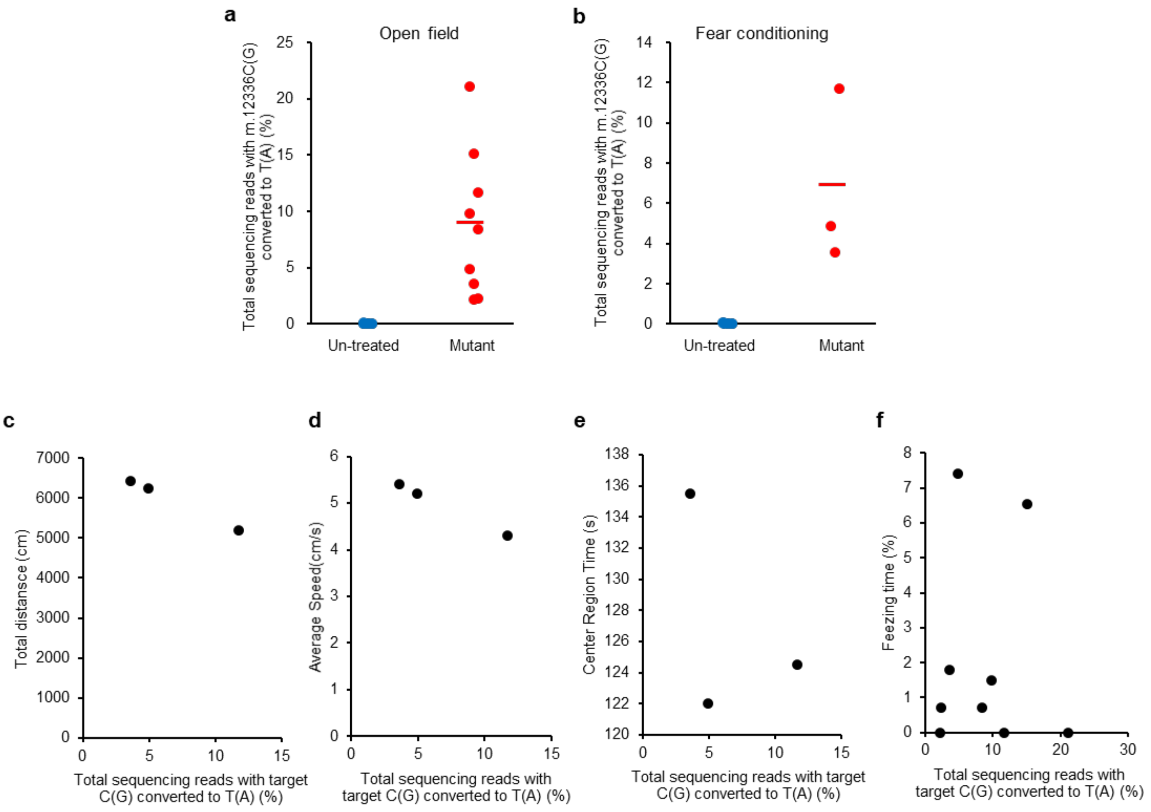

Supplementary Fig. 6. Correlations between editing efficiency and results of behavioral experiments in mutant mice. a. and **b.** Editing efficiency of target m.C12336T in mice used in open filed test and fear conditioning. Average editing frequency was 6.7 % for mice used in open field test and 8.8 % for mice used in fear conditioning test. Correlations between target m.C12336T editing efficiency and **c.** Total distance (cm), **d.** Average speed (cm/s), **e.** Center stay time (s), and **f.** freezing time in mutant mice.

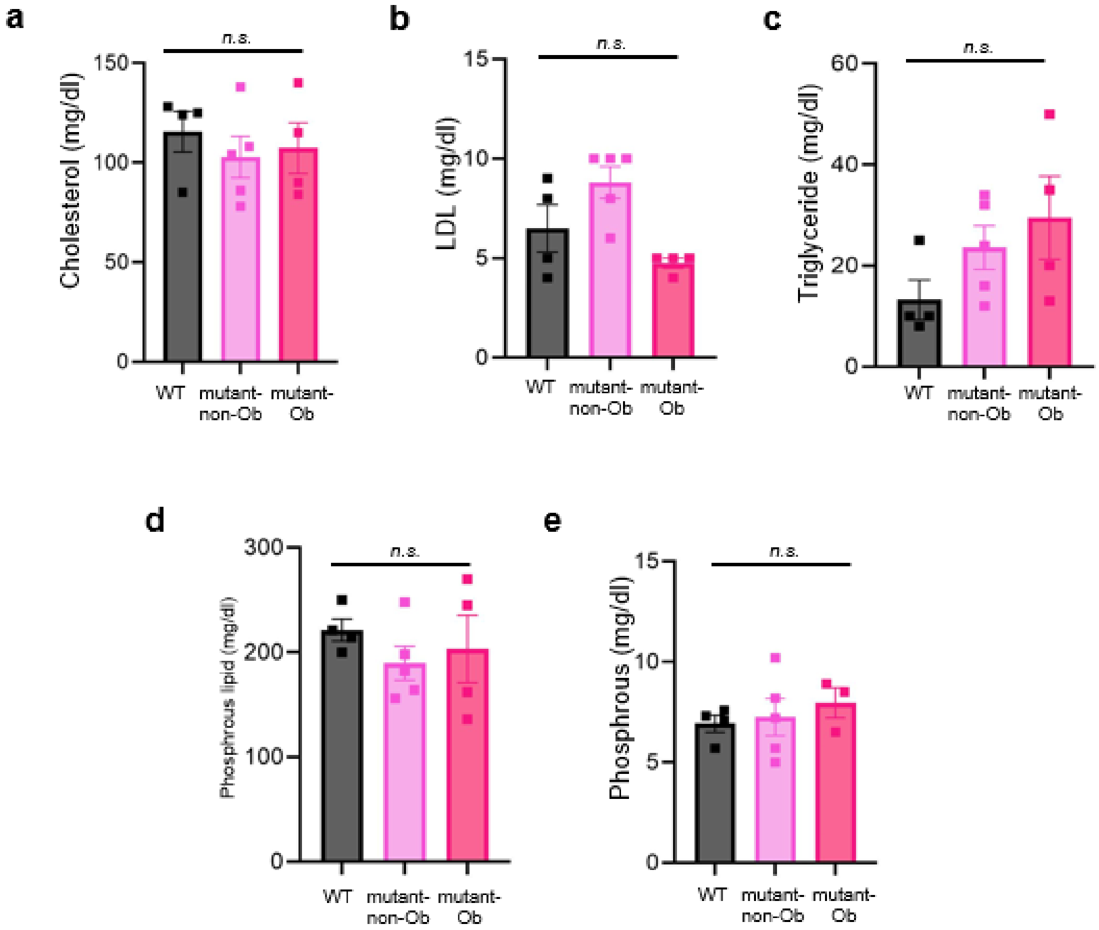

Supplementary Fig. 7. Metabolic bio-marker assessments in sera of wild-type mice (n = 4) and mutant mice (n ≥ 3). Serum levels of **a.** Total Cholesterol, **b.** LDL, **c.** Triglyceride, **d.** Phosphor lipid, and **e.** Phosphorous were measured in WT, mutant-non-Ob, and mutant-Ob mice. Error bars indicate s.e.m. for biologically independent samples (n ≥ 4). Exact p-values for mutant-non-Ob and mutant-Ob were 0.412 and 0.632 for **(a)** Cholesterol, 0.164 and 0.201 for **(b)** LDL, 0.119 and 0.125 for **(c)** Triglyceride, 0.150 and 0.614 for **(d)** Phosphorous lipid, 0.522 and 0.551 for **(e)** Phosphorous (**p* < 0.05, ***p* < 0.01, ****p* < 0.001 and *n.s.* not significant, using Student's two-tailed t-test).

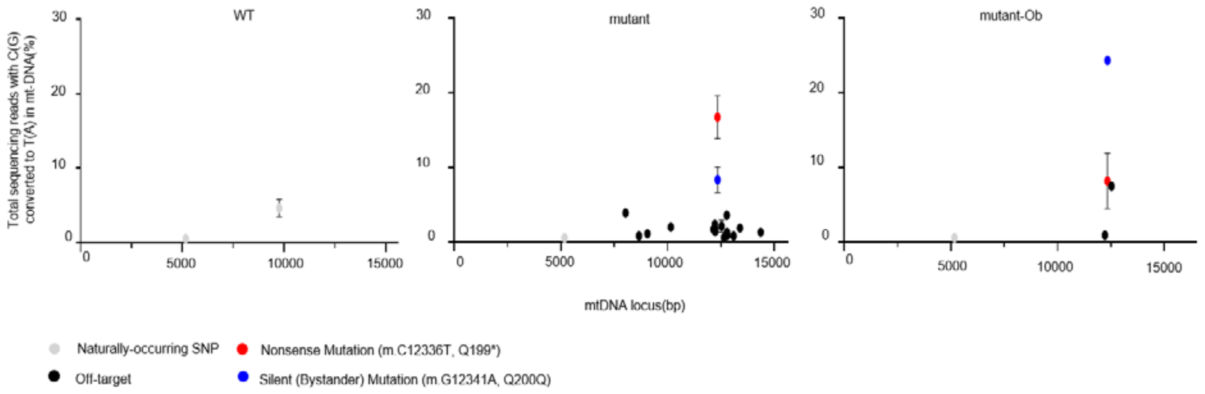

Supplementary Fig. 8. Off-target activities induced by DdCBE in whole mitochondrial genome of wild-type, mutant-non-Ob, and mutant-Ob mice. Mitochondrial genome-wide off-target editing in WT and mutant mice. On-target editing efficiency, silent bystander editing, naturally-occurring SNP, and off-target are shown in red, blue, grey, and black, respectively. Data are shown as means. Error bars indicate s.e.m. for biologically independent samples (*n* = 3).

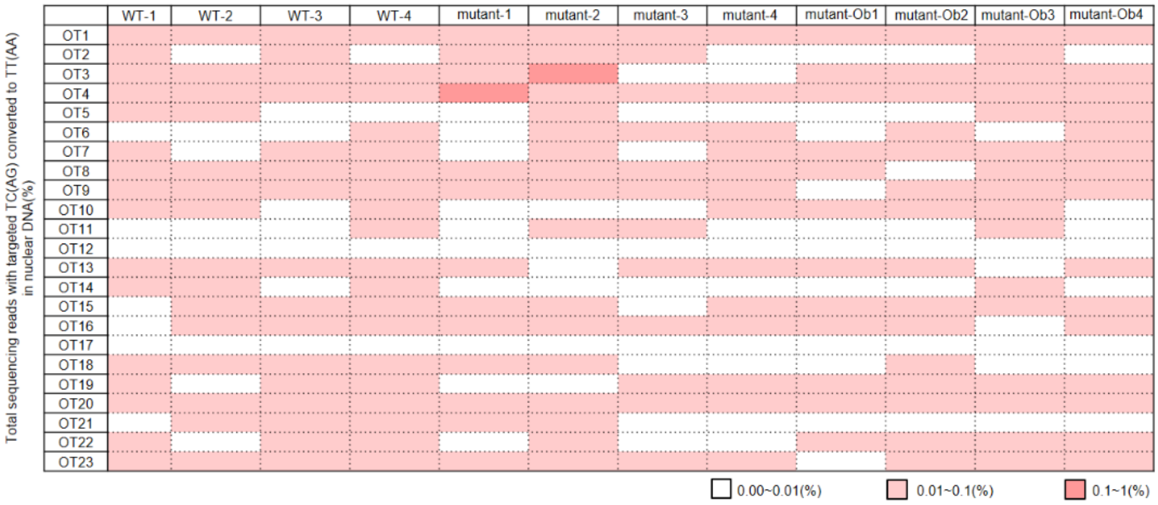

Supplementary Fig. 9. Off-target activities induced by DdCBE in nuclear genome of wild-type, mutant-non-Ob, and mutant-Ob mice. Heatmap depicting off-target activity in nuclear genome regions. Mismatch sites were selected by without or with a single nucleotide mismatch between the left and right TALE-binding DNA sequences.
